# Deciphering molecular overlaps between COPD and NSCLC subtypes (LUAD and LUSC): An integrative bioinformatics study

**DOI:** 10.1097/MD.0000000000043906

**Published:** 2025-08-15

**Authors:** Sevinç Akçay

**Affiliations:** a Department of Molecular Biology and Genetics, Kirşehir Ahi Evran University, Faculty of Art and Sciences, Kirşehir, Turkey.

**Keywords:** bioinformatics, COPD, drug repurposing, gene regulatory networks, LUAD, LUSC

## Abstract

Chronic obstructive pulmonary disease (COPD) and non-small-cell lung cancer (NSCLC) are major global health problems. Despite clinical differences, emerging evidence suggests common molecular underpinnings, particularly between COPD and the 2 primary NSCLC subtypes, lung adenocarcinoma (LUAD) and lung squamous cell carcinoma (LUSC). In this integrative bioinformatics case-control study, we analyzed gene expression datasets (GSE76925, GSE18842, and GSE10072) obtained from the Gene Expression Omnibus (GEO) database, each comprising case-control samples. Differentially expressed genes (DEGs) were identified in COPD, LUAD, and LUSC patients. We performed analyses of protein–protein interaction (PPI) networks, investigated regulatory networks involving transcription factors (TFs) and microRNAs (miRNAs), and conducted pathway enrichment analysis using Kyoto Encyclopedia of Genes and Genomes (KEGG) and Gene Ontology (GO) databases. Drug-gene interactions were assessed to explore therapeutic opportunities. We identified 15 shared DEGs between COPD, LUAD, and LUSC, with distinct gene expression patterns between COPD-LUAD and COPD-LUSC. Notably, LUAD exhibited stronger molecular similarities with COPD than with LUSC. PPI analysis revealed COL10A1, POSTN, SPP1, MMP11, and GREM1 as key hub genes in extracellular matrix (ECM) remodeling and tumor progression. Functional enrichment highlighted ECM organization, apoptosis regulation, and transcriptional control as crucial processes in COPD-associated NSCLC. COPD-LUAD was enriched in cancer-related pathways, while COPD-LUSCs showed stronger immune and lipid metabolism involvement. Regulatory network analysis identified SP1 as a key transcriptional regulator, particularly in COPD-LUAD cells. miRNA-DEG interactions highlighted “hsa-miR-335-5p,” “hsa-miR-192-5p,” and “hsa-miR-215-5p” as the central regulators of COPD and NSCLC. Drug-gene interaction analysis identified Marimastat, Bosentan, and Minocycline as potential therapeutic agents that target key molecular pathways shared across COPD, LUAD and LUSC. Finally, survival analysis revealed that SPP1 (in LUAD), POSTN, and GREM1 (in LUSC) were significantly associated with better overall survival, suggesting their dual utility as both biomarkers and prognostic indicators. Our research highlights the unique regulatory distinctions between LUAD and LUSC while offering fresh insights into the molecular pathways that COPD and NSCLC share. This study represents the initial effort to map TF-miRNA-DEG-drug networks across COPD and both the LUAD and LUSC subtypes. These results suggest potential biomarkers for early diagnosis and highlight opportunities for drug repurposing to mitigate COPD-associated lung cancer risk.

## 1. Introduction

Lung cancer, especially non-small-cell lung cancer (NSCLC) and chronic obstructive pulmonary disease (COPD), are substantial worldwide health burden with significant disease burden and fatality.^[1]^ COPD is a gradually worsening inflammatory illness defined by prolonged breathing difficulties and reduced airflow capacity. It is frequently caused by sustained exposure to environmental contaminants and cigarette smoking, as well as immunological responses, inflammation, and hereditary factors.^[2,3]^ However, globally, lung cancer results in the greatest number of cancer deaths.^[4]^ Lung cancer is classified into 2 major types: small-cell lung cancer (SCLC) and NSCLC. Among NSCLC cases, the most common subtypes are lung adenocarcinoma (LUAD) and lung squamous cell carcinoma (LUSC).^[5]^

The likelihood of developing lung cancer is 2 to 5 times greater in COPD patients than in individuals without COPD.^[6]^ As NSCLC is the most common form of lung cancer, COPD is considered an independent risk factor, indicating a strong molecular and pathophysiological connection between the 2 conditions.^[7]^ LUSC has been shown to have a strong correlation with COPD.^[8]^ COPD and lung cancer, including NSCLC, share several molecular and genetic characteristics, which suggest a common pathogenic link. Several similar pathways, including aberrant immunity, cell proliferation, apoptosis, and chromatin changes, may cause patients to develop both COPD and lung cancer.^[9]^ Evidence suggests that inflammation in COPD causes the occurrence of lung cancer and that COPD is a risk factor for lung cancer since both diseases share genetic risk factors for smoking-induced inflammation. These studies imply that these 2 diseases are associated with the inflammatory pathways.^[10]^ Similar pathways are triggered in patients with NSCLC and COPD.^[11]^ Despite all of this knowledge, how these 2 illnesses are related is still unknown. While previous studies, including Liao et al, have shown associations between COPD and NSCLC, our study uniquely employs a multi-layered bioinformatics approach to explore transcriptional and post-transcriptional regulatory overlaps.^[12]^

This study utilized an integrative bioinformatics approach to analyze gene expression profiles from 3 publicly available Gene Expression Omnibus (GEO) datasets (GSE76925, GSE18842, and GSE10072). This study sought to uncover common differentially expressed genes (DEGs) associated with COPD, the 2 most common types of NSCLC, and the specific molecular interactions unique to COPD-LUSC and COPD-LUAD. Our analyses included the construction of protein–protein interaction (PPI) networks; functional enrichment analysis using Gene Ontology (GO) and Kyoto Encyclopedia of Genes and Genomes (KEGG) pathways; and regulatory network analysis incorporating transcription factor (TF)-DEG, miRNA-DEG, and miRNA-DEG-TF interactions. Additionally, we performed drug-gene interaction analyses to determine possible therapeutic targets, highlighting opportunities for drug repurposing.

The study’s primary goal was to systematically explore and decipher the molecular interrelationships between COPD and the 2 major subtypes of NSCLC, lung LUAD and LUSC, using an integrative bioinformatics approach. Recognizing that individuals with COPD are far more likely to acquire lung cancer, we sought to identify shared and distinct gene expression signatures, regulatory networks, and pathway-level interactions that could explain this clinical overlap and identify potential biomarkers and therapeutic targets. Through this multifaceted analysis, our goal was to provide a comprehensive molecular framework explaining the higher prevalence of lung cancer among individuals with COPD, highlight distinct regulatory architectures between LUAD and LUSC in the context of COPD, and propose targeted therapeutic strategies that could mitigate this risk and lead to better patient results.

## 2. Materials and methods

### 2.1. GSE datasets acquisition

To identify shared DEGs, TFs, and microRNA (miRNAs) among COPD, NSCLC, and lung cancer, we retrieved freely available gene expression profiles acquired the GEO database and the repository maintained by the National Center for Biotechnology Information (NCBI). Gene expression profiles from 3 3 datasets, GSE76925, GSE18842, and GSE10072, were selected based on their relevance, sample size, and data quality. There were 111 COPD patients and 40 controls in the GSE76925 dataset, 32 LUSC patients and 45 controls in the GSE18842 dataset, and 58 LUAD patients and 49 controls in the GSE10072 dataset. Table 1 provides comprehensive details of the samples.^[13–15]^ There were no human or animal participants in this study, which made use of publicly accessible datasets from the GEO database. Thus, informed permission and an ethical review were not necessary.

**Table 1:** The GEO datasets that we examined and utilized for this research.

Dataset	Disease	Tissue type	Control Group, n	Case Group, n	Method	Platform
GSE76925	COPD	Lung tissue	40	111	“Expression profiling by array”	“Illumina HumanHT-12 V4.0 expression beadchip”
GSE18842	LUSC	Lung tissue	32	46	“Expression profiling by array”	“Affymetrix Human Genome U133 Plus 2.0 Array”
GSE10072	LUAD	Lung tissue	49	58	“Expression profiling by array”	“Affymetrix Human Genome U133A Array”

COPD = chronic obstructive pulmonary disease, LUAD = lung adenocarcinoma, LUSC = lung squamous cell carcinoma.

### 2.2. Identification of common DEGs

**Figure 1.**
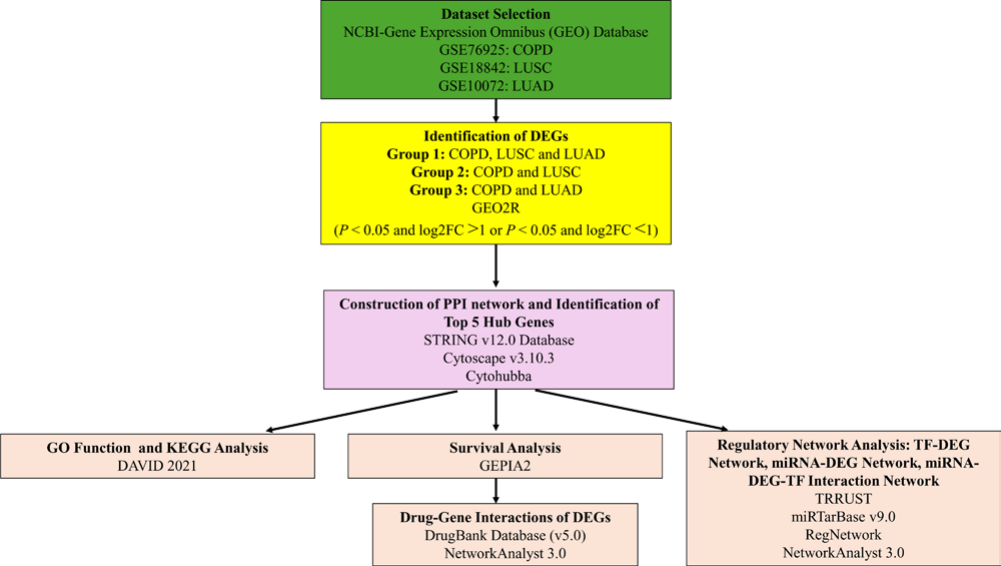
Workflow diagram of the bioinformatics analysis conducted to identify common molecular signatures among COPD, LUSC, and LUAD. COPD = chronic obstructive pulmonary disease, DAVID = Database for Annotation, Visualization, and Integrated Discovery, DEG = differentially expressed gene, GEO = Gene Expression Omnibus, GEPIA, 2 = gene expression profiling interactive analysis 2, GO = gene ontology, KEGG = Kyoto Encyclopedia of Genes and Genomes, LUAD = lung adenocarcinoma, LUSC = lung squamus cell carcinoma, miRNA = microRNA, PPI = protein–protein interaction, STRING = search tool for the retrieval of interacting genes/proteins, TF = transcription factor, TRRUST = transcriptional regulatory relationships unraveled by sentence-based text mining.

DEGs were classified into 3 groups: Group 1: COPD, LUSC, and LUAD; Group 2: COPD and LUSC; and Group 3: COPD and LUAD by the GEO2R statistical tool.^[16]^ *P* < .05, log2FC > 1 (upregulated DEGs), or *P* < .05, and log2FC < 1 (downregulated DEGs) were the criteria used to identify DEGs. Overrepresentation of protein categories was observed using the PANTHER database. Venn diagrams and heatmaps were generated to visualize the overlapping DEGs across the groups. Figure 1 illustrates the steps involved in our study.

### 2.3. Building a PPI network and determining the top 5 hub genes

To investigate how the shared differentially expressed genes (DEGs) interact, a protein-protein interaction (PPI) network was created using the Search Tool for the Retrieval of Interacting Genes/Proteins (STRING) database.^[17]^ The following criteria were applied: confidence score > 0.4 (medium confidence interactions). The network was graphically represented using the Cytoscape software (v3.10.3). CytoHubba, a Cytoscape plug-in, was used to identify hub genes by ranking them according to their importance within the PPI network.^[18]^ The 5 most important hub genes within each group were ranked using the Maximum Clique Centrality (MCC) algorithm.

### 2.4. Functional analysis of common DEGs

The Database for Annotation, Visualization, and Integrated Discovery (DAVID) was employed to perform GO enrichment analysis, classifying DEGs into Biological Process (BP)**–**cellular and molecular activities influenced by DEGs, Cellular Component (CC)–localization of DEGs within the cell, and Molecular Function (MF)–molecular activities associated with DEGs.^[19]^ *P* < .05 criterion was used to identify significantly enriched terms. To identify the biological pathways connected to DEG, a KEGG pathway enrichment analysis was carried out. A *P*-value below 0.05 was used as the criterion for statistical significance.

### 2.5. Regulatory network analysis: TF-DEG network, miRNA-DEG network, miRNA-DEG-TF interaction network

The Transcriptional Regulatory Relationships Unraveled by Sentence-based Text Mining (TRRUST v2.0) database was used to identify TFs that control common DEGs.^[20]^ A network showing interactions between TFs and DEGs was built using NetworkAnalyst, a tool for visualizing regulatory networks. The miRNA Data Integration Portal (mirDIP v4.1), which combines information from 30 different miRNA databases, was used to predict miRNA interactions with DEGs.^[21]^ To investigate both post-transcriptional and transcriptional controls, miRNA-DEG-TF interaction networks were constructed. Networks were compared across disease groups to identify key regulatory elements using NetworkAnalyst.^[22]^

### 2.6. Drug-DEG network analysis

We used the DrugBank database to identify potential treatment targets for the top hub genes found in our analysis, and the DEG-TF-miRNA-drug network was constructed using the NetworkAnalyst tool. This network highlights key druggable targets and potential drug repurposing opportunities for COPD-associated lung cancer. The DrugBank database provides information on these medications (https://go.drugbank.com/).

### 2.7. Survival analysis

Survival analysis was conducted to evaluate whether the expression levels of the 5 most central hub genes correlated with patient prognosis in LUAD and LUSC (http://gepia2.cancer-pku.cn/#survival). Kaplan–Meier plots were created to visually compare the overall survival rates between patient groups exhibiting high versus low expression levels of the genes. Log-rank tests were applied to ascertain the statistical significance of differences in survival rates, with a *P*-value under 0.05 being the threshold for significance. Analyses were conducted using the default GEPIA2 parameters.

## 3. Results

### 3.1. Identification of common DEGs

**Figure 2.**
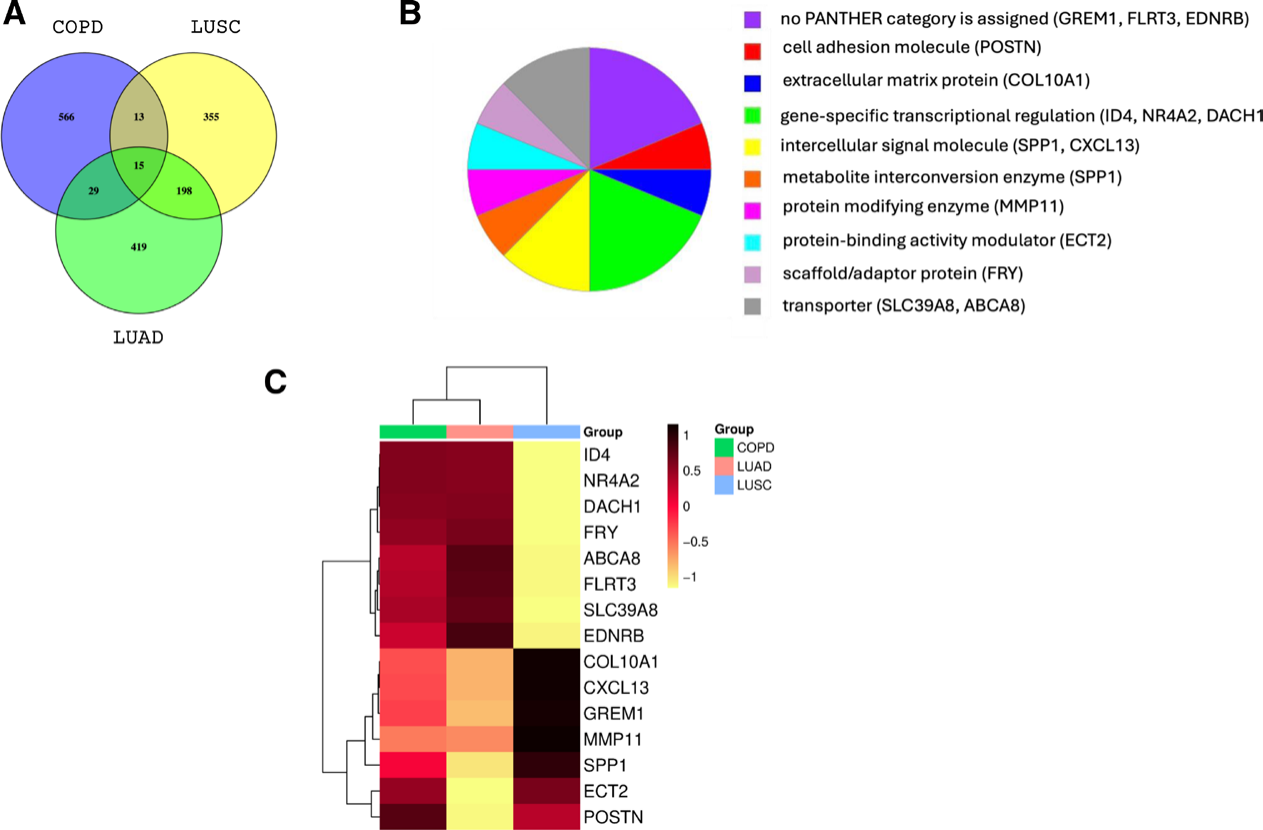
(A) Venn diagram of common DEGs in COPD, LUSC, and LUAD, (B) Sorting common DEGs according to the PANTHER protein class in COPD, LUSC, and LUAD, (C) Heatmap of 15 common DEGs in COPD (green), LUSC (blue) and LUAD (pink). The color intensity indicates expression levels. COPD = chronic obstructive pulmonary disease, DEG = differentially expressed gene, LUAD = lung adenocarcinoma, LUSC = lung squamus cell carcinoma.

To identify the shared molecular mechanisms among COPD, LUSC, and LUAD, we analyzed gene expression profiles from 3 datasets (GSE76925, GSE18842, and GSE10072). Venn diagrams were generated to visually represent the overlap of DEGs among the 3 disease groups (Fig. 2A). Fifteen common DEGs were identified in COPD, LUSC, and LUAD. The complete list of DEGs shared across COPD, LUSC and LUAD is presented in Table 2. Thirteen common DEGs were identified between the COPD and LUSC groups. The complete list of DEGs shared between COPD and LUSC is presented in Table S1 (Supplemental Digital Content, https://links.lww.com/MD/P670). 29 common DEGs were identified in the patients with COPD and LUAD. The complete list of DEGs shared between COPD and LUAD is presented in Table S2 (Supplemental Digital Content, https://links.lww.com/MD/P670). The PANTHER database was used to classify the identified DEGs into functional categories. DEGs shared across COPD, LUSC, and LUAD (15 genes) were classified into 9 categories: “cell adhesion molecule,” “extracellular matrix protein,” “gene-specific transcriptional regulator,” “intercellular signal molecule,” “metabolite interconversion enzyme,” “protein modifying enzyme,” “protein-binding activity modulator,” “scaffold/adaptor protein” and “transporter” (Fig. 2B). A heatmap of the 15 common DEGs in COPD, LUSC, and LUAD is shown in Figure 2C.

**Table 2:** Full List of Common Differentially Expressed Genes (DEGs) between COPD, LUSC and LUAD.

Gene symbol	COPD		LUSC		LUAD	
	*P*-value	Log2FC	*P*-value	Log2FC	*P*-value	Log2FC
ABCA8	1.21e−05	1.73948	1.88e−33	−2.01619	4.47e−38	2.95990
DACH1	5.18e−08	1.43870	7.35e−24	−1.24543	2.32e−26	1.44287
ECT2	1.14e−04	1.08303	1.28e−34	1.31460	1.17e−19	−1.66432
EDNRB	2.34e−08	1.65585	6.09e−36	−1.56849	8.06e−40	3.16126
FLRT3	9.77e−09	1.025921	2.88e−18	−1.07420	2.55e−14	1.61339
FRY	1.02e−06	1.723731	1.16e−30	−1.06821	1.16e−26	1.94567
ID4	9.16e−05	1.95111	9.83e−30	−1.53539	3.69e−25	1.90987
NR4A2	2.04e−03	1.14356	4.80e−17	−1.04705	4.51e−06	1.10518
POSTN	1.70e−04	1.82271	2.64e−14	1.09037	5.90e−13	−1.32028
SLC39A8	2.11e−02	1.17397	7.63e−28	−1.15961	1.32e−20	1.64111
COL10A1	7.35e−05	−1.47929	2.17e−22	1.55908	1.52e−21	−2.46347
CXCL13	6.27e−03	−1.19509	8.38e−10	1.27502	1.32e−13	−2.05430
GREM1	3.31e−04	−1.16387	6.74e−27	2.18399	8.05e−25	−2.54836
MMP11	6.38e−05	−1.31976	6.38e−24	1.14627	5.65e−17	−1.42853
SPP1	4.68e−05	−1.30678	1.23e−19	1.37565	5.22e−40	−4.36441

COPD = chronic obstructive pulmonary disease, DEG = differentially expressed gene, LUAD = lung adenocarcinoma, LUSC = lung squamous cell carcinoma.

### 3.2. Building a PPI network and determining the top 5 hub genes

**Figure 3.**
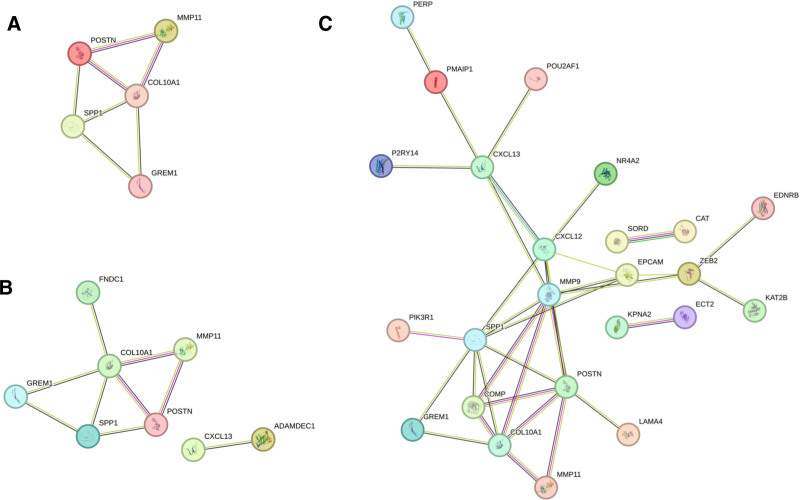
(A) PPI network constructed using common DEGs identified across COPD, LUSC, and LUAD, (B) PPI network of DEGs commonly dysregulated between COPD and LUSC, (C) PPI network of DEGs shared between COPD and LUAD. COPD = chronic obstructive pulmonary disease, DEG = differentially expressed gene, LUAD = lung adenocarcinoma, LUSC = lung squamus cell carcinoma, PPI = protein–protein interaction.

For each dataset, a statistically significant PPI network was built by analyzing the common DEGs within the STRING database. Cytoscape was employed for network visualization and Cytohubba was used for topological calculations. The PPI values of COPD-LUSC-LUAD (*P *= 7.25e-05), COPD-LUSC (*P* = .00041), and COPD-LUAD (*P* = 4.14e-07) were found, and a statistically significant PPI network was created. Statistical significance was assessed using STRING interaction confidence scores (>0.4) and enrichment p-values (*P* < .05) calculated during network generation. Figure 3A–C illustrates the PPI networks of the DEGs identified in COPD, LUSC, and LUAD.

The CytoHubba plug-in within Cytoscape was used to examine the structural features of the PPI network, and the MCC algorithm was applied to pinpoint the 5 most central hub genes within each group. The top 5 hub genes in COPD-LUSC-LUAD were COL10A1, POSTN, SPP1, MMP11, and GREM1. The top 5 hub genes in COPD-LUAD based on the MCC term were MMP9, EPCAM, ZEB2, CXCL12, and CAT. No hub genes were found in COPD-LUSCs based on the MCC term.

### 3.3. Functional analysis of common DEGs

**Figure 4.**
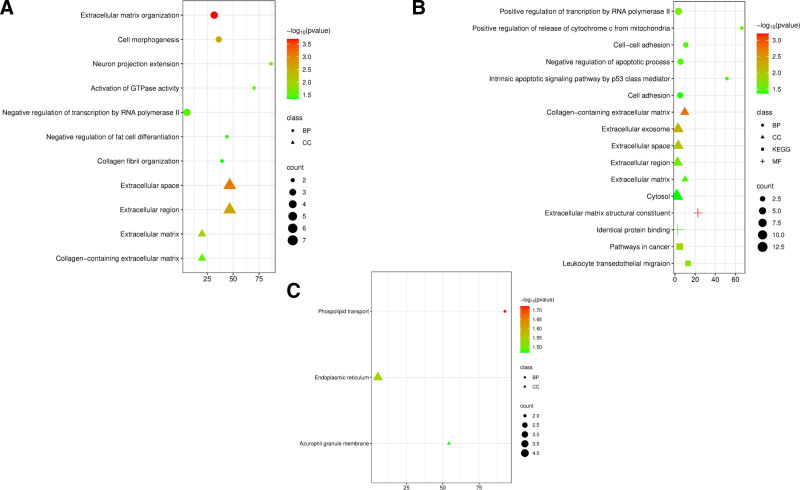
Functional enrichment analysis of the common DEGs (A) in COPD, LUSC and LUAD (B) COPD and LUAD, (C) COPD and LUSC. The circle represents BP, the triangle indicates CC, the square indicates the MF term, the plus indicates KEGG term. The color indicates the *P*-values, and the size of the shapes indicates the gene number. The − log10 (*P*-value) indicates the statistical significance of the enrichment, where higher values correspond to greater significance. The count of genes is represented by circle size. BP = biological process, CC = cellular component, COPD = chronic obstructive pulmonary disease, DEG = differentially expressed gene, KEGG = Kyoto Encyclopedia of Genes and Genomes, LUAD = lung adenocarcinoma, LUSC = lung squamus cell carcinoma, MF = molecular function.

All DEGs were characterized using DAVID according to their GO and KEGG pathway BPs, MFs, and CCs. The GO terms between COPD, LUSC, and LUAD in the BP and CC categories are shown in Figure 4A. In the BP category, GO terms were “extracellular matrix organization,” “cell morphogenesis,” “neuron projection extension,” “negative regulation of transcription RNA polymerase II,” “negative regulation of fat cell differentiation,” and “Collagen fibril organization.” The most popular GO terms within the CC category were “extracellular space,” “extracellular matrix,” “collagen-containing extracellular matrix,” and “extracellular region.” The GO terms for COPD and LUSC are shown in Figure 4B. The only GO term within BP category was’ “phospholipid transport.” The top GO terms of the CC category were “endoplasmic reticulum,” “azurophil granule membrane,” and “plasma membrane.” The GO terms and KEGG pathways for COPD and LUAD are shown in Figure 4C. The top GO terms within the BP category were “positive regulation of transcription by RNA polymerase II,” “positive regulation of cytochrome c release from mitochondria,” “cell–cell adhesion,” “negative regulation of apoptotic process,” and “intrinsic apoptotic signaling pathway by p53 class mediator.” Within the CC category, the most popular GO terms are “extracellular exosome,” “collagen-containing extracellular matrix,” “extracellular space,” “extracellular region,” and “extracellular matrix.” Within the MF category, the GO terms were “extracellular matrix structural constituent,” “identical protein binding,” and “collagen binding.” Key KEGG pathways are “pathways in cancer” “leukocyte transendothelial migration,” “focal adhesion” and “viral carcinogenesis.”

### 3.4. Regulatory network analysis: TF-DEG network, miRNA-DEG network, miRNA-DEG-TF interaction network

**Figure 5.**
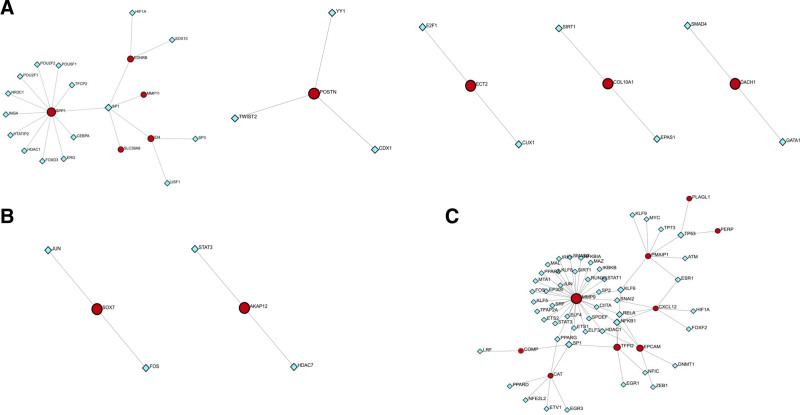
TF-DEG interaction networks (A) COPD, LUSC, and LUAD, (B) COPD and LUSC network, (C) COPD and LUAD network. Red nodes denote DEGs, while blue squares represent TFs. COPD = chronic obstructive pulmonary disease, DEG = differentially expressed gene, LUAD = lung adenocarcinoma, LUSC = lung squamus cell carcinoma, TF = transcription factor.

**Figure 6.**
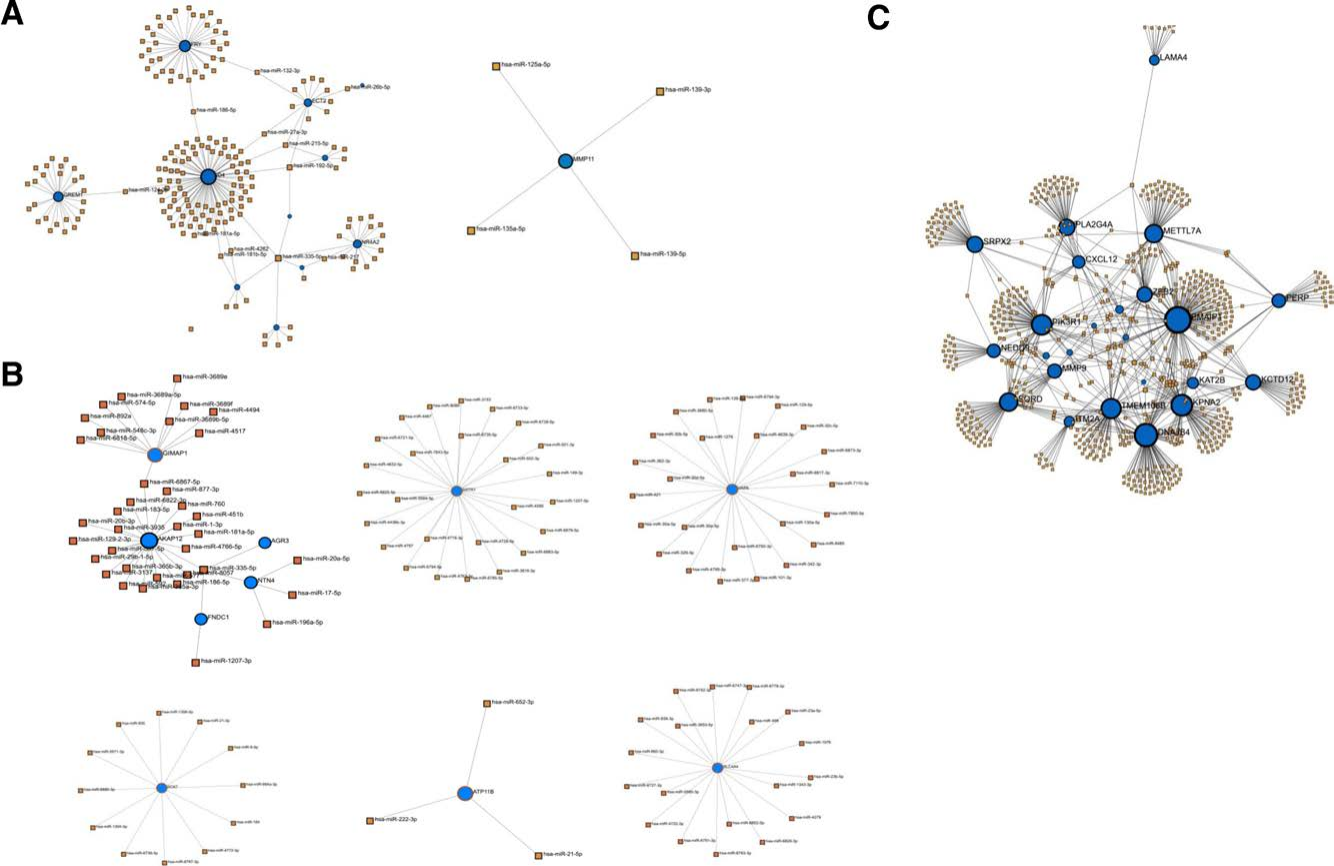
miRNA-DEG interaction networks (A) miRNA-DEG interaction network for COPD, LUSC, and LUAD, (B) miRNA-DEG interaction network for COPD and LUSC, (C) miRNA-DEG interaction network for COPD and LUAD. Blue nodes represent DEGs, while brown nodes represent miRNAs. COPD = chronic obstructive pulmonary disease, DEG = differentially expressed gene, LUAD = lung adenocarcinoma, LUSC = lung squamus cell carcinoma.

**Figure 7.**
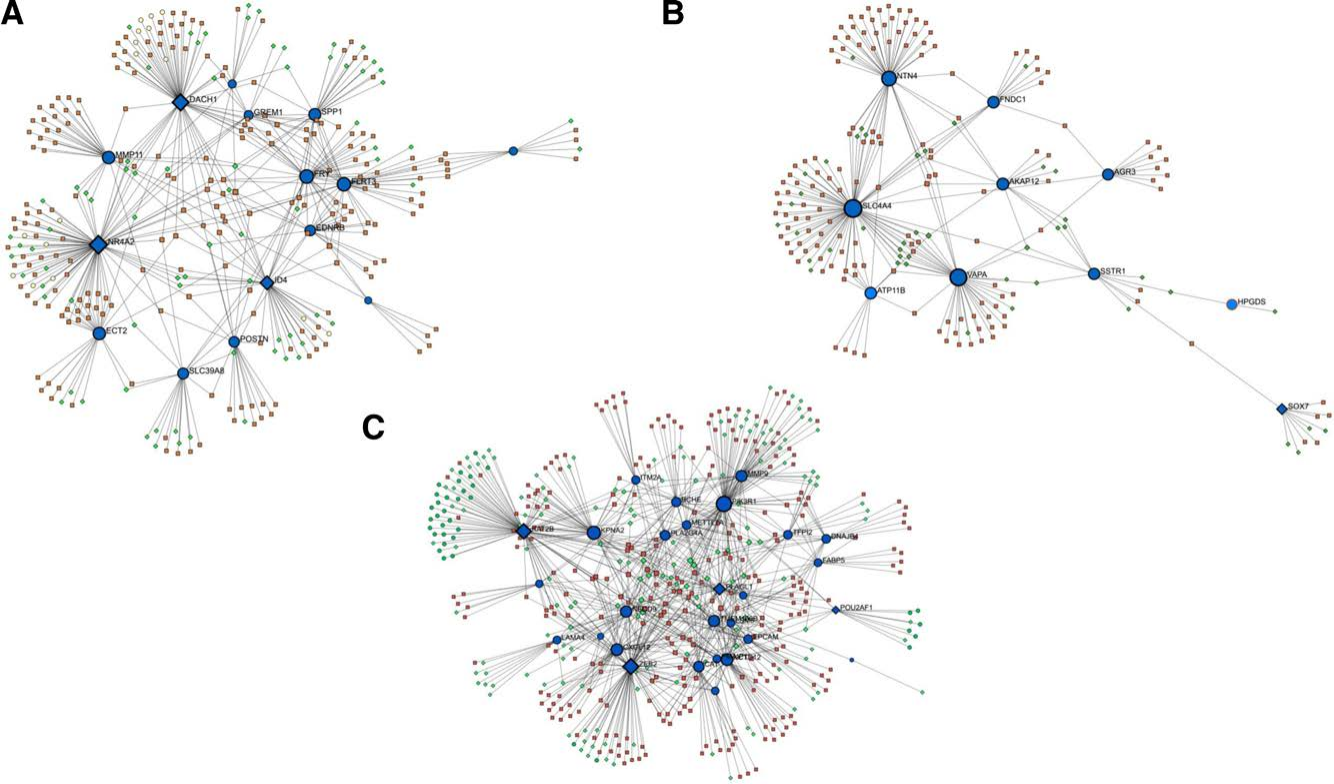
miRNA-DEG-TF interaction networks. (A) miRNA-DEG-TF interaction network for COPD, LUSC, and LUAD, (B) miRNA-DEG-TF interaction network for COPD and LUSC, (C) miRNA-DEG-TF interaction network for COPD and LUAD. Blue nodes represent DEGs, green nodes represent TFs and brown nodes represent miRNAs. COPD = chronic obstructive pulmonary disease, DEG = differentially expressed gene, LUAD = lung adenocarcinoma, LUSC = lung squamus cell carcinoma, TF = transcription factor.

To further investigate the molecular mechanisms, we constructed networks for TF-DEG, miRNA-DEGs, and miRNA-DEG-TFs in 3 groups: COPD, LUSC, LUAD, COPD and LUSC, and COPD and LUAD (Figs. 5–7). We first identified the TFs that regulated common DEGs. SP1 emerged as the most prominent TF, regulating 5 DEGs including SPP1, SLC39A8, ID4, MMP11, and EDNR8. Among these, SPP1 was targeted by the highest number of TFs (Fig. 5A). The COPD-LUSC network (Fig. 5B) was less interconnected, suggesting distinct transcriptional regulation, with SOX7 regulated by JUN and FOS, and AKAP12 by STAT3 and HDAC7. In contrast, the COPD-LUAD network (Fig. 5C) was more densely connected, with SP1 and NFKB1 each regulating 4 DEGs, and MMP9 identified as a major hub regulated by 33 TFs. The complete list of TFs regulating shared DEGs is provided in Table S3 (Supplemental Digital Content, https://links.lww.com/MD/P670).

Figure 6A presents the miRNA-DEG interaction network shared among COPD, LUSC, and LUAD, highlighting 12 miRNAs that target at least 2 DEGs. *ID4* had the highest number of targeting miRNAs, followed by FRY (38), GREM1 (26), NR4A2 (16), and ECT2 (14). The complete list of shared miRNAs is detailed in Table 3.

**Table 3:** Key miRNAs identified in the regulatory network analysis.

miRNA name	Number of target genes	Associated group(s)	miRNA name	Number of target genes	Associated group(s)
“hsa-miR-335-5p”	6	COPD, LUAD, LUSC	“hsa-miR-30a-5p”	3	COPD, LUAD
“hsa-miR-192-5p”	4	COPD, LUAD, LUSC	“hsa-miR-5011-5p”	3	COPD, LUAD
“hsa-miR-215-5p”	3	COPD, LUAD, LUSC	“hsa-miR-190a-3p”	3	COPD, LUAD
“hsa-miR-186-5p”	2	COPD, LUAD, LUSC	“hsa-miR-146a-5p”	3	COPD, LUAD
“hsa-miR-124-3p”	2	COPD, LUAD, LUSC	“hsa-miR-448”	3	COPD, LUAD
“hsa-miR-132-3p”	2	COPD, LUAD, LUSC	“hsa-miR-6747-3p”	3	COPD, LUAD
“hsa-miR-27a-3p”	2	COPD, LUAD, LUSC	“hsa-miR-6778-3p”	3	COPD, LUAD
“hsa-miR-181a-5p”	2	COPD, LUAD, LUSC	“hsa-miR-320a”	3	COPD, LUAD
“hsa-miR-181b-5p”	2	COPD, LUAD, LUSC	“hsa-miR-297”	3	COPD, LUAD
“hsa-miR-4262”	2	COPD, LUAD, LUSC	“hsa-miR-144-3p”	3	COPD, LUAD
“hsa-miR-26b-5p”	2	COPD, LUAD, LUSC	“hsa-miR-29b-3p”	3	COPD, LUAD
“hsa-miR-217”	2	COPD, LUAD, LUSC	“hsa-miR-302a-5p”	3	COPD, LUAD
“hsa-miR-335-5p”	4	COPD, LUSC	“hsa-miR-20a-5p”	3	COPD, LUAD
“hsa-miR-6867-5p”	2	COPD, LUSC	“hsa-miR-106a-5p”	3	COPD, LUAD
“hsa-miR-16-5p”	6	COPD, LUAD	“hsa-miR-20b-5p”	3	COPD, LUAD
“hsa-miR-17-5p”	6	COPD, LUAD	“hsa-miR-526b-3p”	3	COPD, LUAD
“hsa-miR-26b-5p”	6	COPD, LUAD	“hsa-miR-519d-3p”	3	COPD, LUAD
“hsa-miR-10a-5p”	5	COPD, LUAD	“hsa-miR-142-3p”	3	COPD, LUAD
“hsa-miR-192-5p”	5	COPD, LUAD	“hsa-miR-181a-5p”	3	COPD, LUAD
“hsa-miR-215-5p”	5	COPD, LUAD	“hsa-miR-222-3p”	3	COPD, LUAD
“hsa-miR-124-3p”	4	COPD, LUAD	“hsa-miR-15b-5p”	3	COPD, LUAD
“hsa-miR-3924”	4	COPD, LUAD	“hsa-miR-1273g-3p”	2	COPD, LUAD
“hsa-miR-221-3p”	4	COPD, LUAD	“hsa-miR-29a-5p”	2	COPD, LUAD
“hsa-miR-155-5p”	4	COPD, LUAD	“hsa-miR-4307”	2	COPD, LUAD
“hsa-miR-93-5p”	4	COPD, LUAD	“hsa-miR-939-3p”	2	COPD, LUAD
“hsa-miR-106b-5p”	4	COPD, LUAD	“hsa-miR-6849-3p”	2	COPD, LUAD
“hsa-miR-335-5p”	4	COPD, LUAD	“hsa-miR-125b-5p”	2	COPD, LUAD
“hsa-miR-181b-5p”	4	COPD, LUAD	“hsa-miR-4755-3p”	2	COPD, LUAD
“hsa-miR-98-5p”	3	COPD, LUAD	“hsa-miR-3609”	2	COPD, LUAD
“hsa-miR-4738-3p”	3	COPD, LUAD	“hsa-miR-548ah-5p”	2	COPD, LUAD
“hsa-miR-146b-5p”	3	COPD, LUAD	“hsa-miR-342-3p”	2	COPD, LUAD
“hsa-miR-302c-3p”	3	COPD, LUAD	“hsa-let-7e-5p”	2	COPD, LUAD

COPD = chronic obstructive pulmonary disease, DEG = differentially expressed gene, LUAD = lung adenocarcinoma, LUSC = lung squamous cell carcinoma.

Figure 6B illustrates the miRNA-DEG interaction network shared between COPD and LUSC. Notably, “hsa-miR-335-5p” targets 4 DEGs, and “hsa-miR-6867-5p” targets 2. Among the genes, SSTR1 is targeted by the highest number of miRNAs. VAPA, AKAP12, SLC4A4, GIMAP1, and SOX7 are also highly targeted, with 25, 22, 19, 11, and 12 miRNAs respectively. The complete list of shared miRNAs is available in Table 3.

Figure 6C shows the miRNA-DEG interaction network for COPD and LUAD, showing a highly interconnected regulatory network. 39 miRNAs were identified targeting at least 3 DEGs. The full list of shared miRNAs identified across COPD and LUAD is provided in Table 3.

To identify shared transcriptional and post-transcriptional regulatory mechanisms, we constructed an integrated miRNA-DEG-TF network (Fig. 7A). The network revealed high connectivity, with common TFs and miRNAs regulating multiple DEGs across COPD, LUSC, and LUAD. SP1 and CTCF each targeted 3 DEGs, while 21 other TFs regulated 2 DEGs. NR4A2 emerged as a central regulatory node, targeted by the highest number of miRNAs and TFs. Additionally, 19 miRNAs were identified as regulators of at least 3 DEGs. Full lists of TFs and miRNAs are provided in Tables S4 and S5 (Supplemental Digital Content, https://links.lww.com/MD/P670).

In the COPD-LUSC group, SLC4A4 was the most regulated gene, targeted by the highest number of miRNAs and TFs (Fig. 7B). EGR1, USF1, and EGR3 were the most active TFs, with 15 others each targeting 2 DEGs. Among miRNAs, “hsa-miR-145” targeted 4 DEGs, while “hsa-miR-410” and *“*hsa-miR-203” each targeted 3. Additionally, 29 miRNAs were found to regulate 2 DEGs. Detailed lists are available in Tables S4 and S5 (Supplemental Digital Content, https://links.lww.com/MD/P670).

In the COPD-LUAD group, ZEB2 was the most heavily regulated gene, targeted by the highest number of miRNAs and TFs (Fig. 7C). TP53, SP1, and JUN each regulated 7 DEGs, followed by CTCF (6 DEGs) and 6 other TFs (e.g., SMAD3, MYC, USF1) each targeting 5. Among miRNAs, “hsa-miR-222” and “hsa-miR-548c-3p” each targeted 6 DEGs, with several others targeting 5 or 4. Full details are available in Tables S4 and S5 (Supplemental Digital Content, https://links.lww.com/MD/P670).

### 3.5. Drug-DEG network analysis

**Figure 8.**
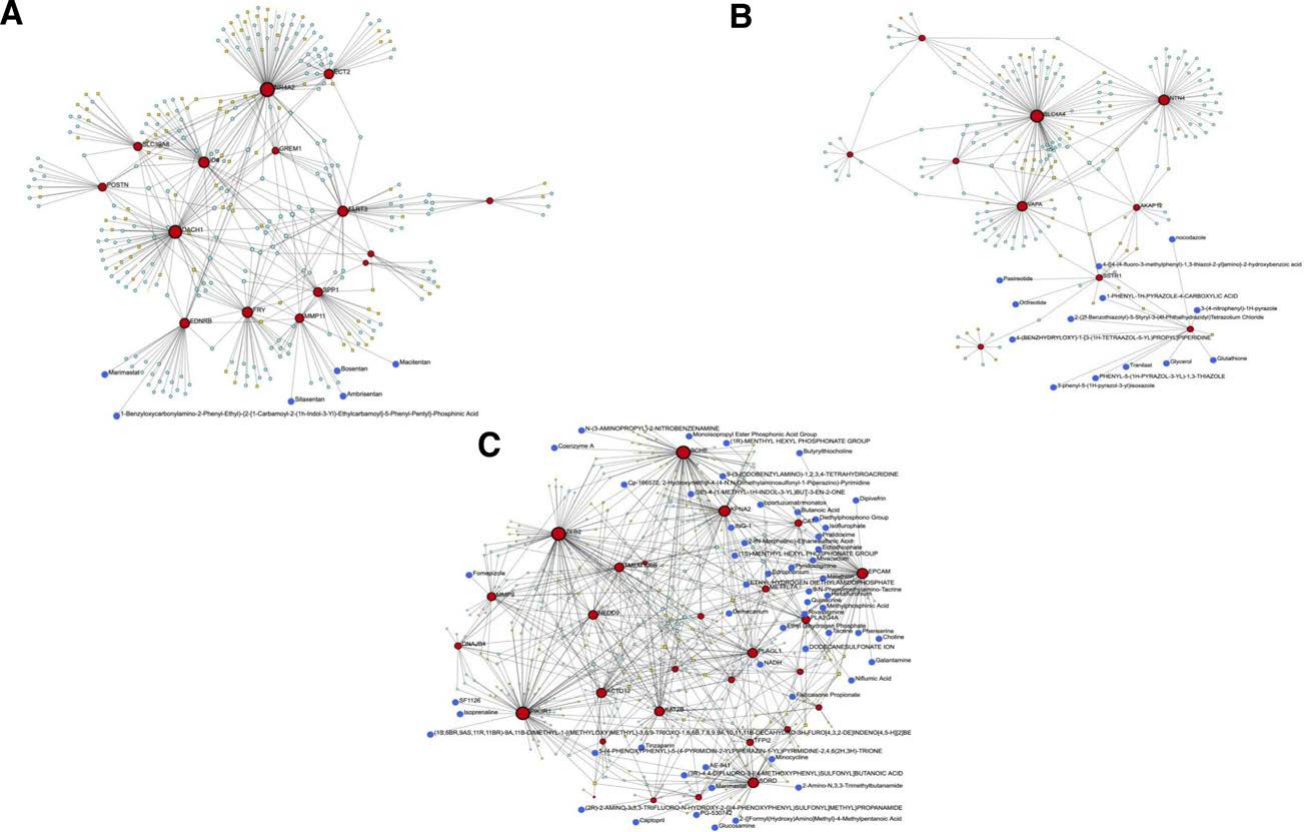
Drug-gene interaction networks (A) COPD, LUSC, and LUAD, (B) COPD and LUSC, (C) COPD and LUAD. Red nodes representing DEGs, yellow nodes representing TFs, light blue nodes representing miRNAs, dark blue nodes representing drugs. COPD = chronic obstructive pulmonary disease, DEG = differentially expressed gene, LUAD = lung adenocarcinoma, LUSC = lung squamus cell carcinoma, TF = transcription factor.

We constructed a TF-DEG-miRNA-drug network to identify possible therapeutic candidates that target important genes linked to LUAD, LUSC, and COPD. Figure 8A shows the shared drug-target interactions across COPD, LUSC, and LUAD. The MMP11(Bosentan, Ambrisentan, Macitentan, and Sitaxentan) and EDNR1 (Marimastat and Benzyloxycarbonylamino) genes have the highest potential drug targets. Figure 8B shows the network highlighting therapeutic targets specific to COPD and LUSC. As shown in Figure 8C, a highly interconnected network was constructed between COPD and LUAD patients. Table 4 provides detailed information about potential drugs.

**Table 4:** Identified drug compounds targeting DEGs in COPD, LUSC, and LUAD, COPD and LUSC, and COPD, and LUAD.

Groups	Drug name	Target gene	DrugBank ID
COPD, LUSC, and LUAD	Bosentan	EDNRB	DB00559
	Sitaxentan	EDNRB	DB06268
	Marimastat	MMP11	DB00786
COPD and LUSC	Octreotide	SSTR1	DB00104
	Pasireotide	SSTR1	DB06663
	Glutathione	HPGDS	DB00143
COPD and LUAD	Choline	BCHE	DB00122
	Tacrine	BCHE	DB00382
	Marimastat	MMP9	DB00786
	Minocycline	MMP9	DB01017
	Fluticasone Propionate	PLA2G4A	DB00588
	Quinacrine	PLA2G4A	DB01103
	SF1126	PIK3R1	DB01064
	Coenzyme A	KAT2B	DB01992
	NADH	SORD	DB00157
	Oportuzumab monatox	EPCAM	DB05319
	ING-1	EPCAM	DB05831

COPD = chronic obstructive pulmonary disease, DEG = differentially expressed gene, LUAD = lung adenocarcinoma, LUSC = lung squamous cell carcinoma.

### 3.6. Survival analysis

**Figure 9.**
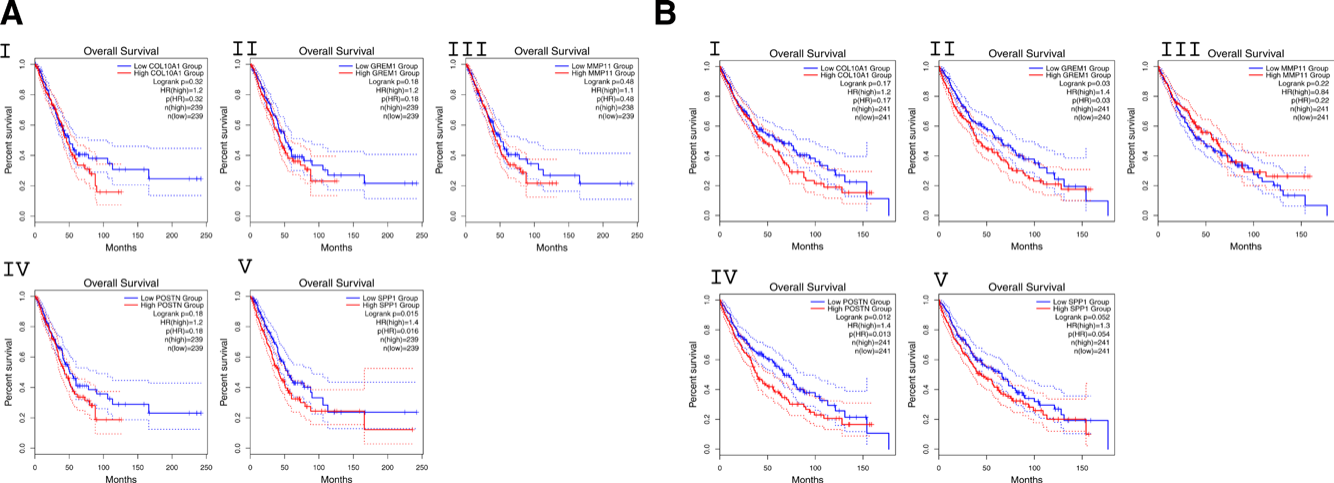
Overall Survival analysis of LUAD and LUSC patients based on GEPIA data. (A) Kaplan–Meier survival analysis conducted with high and low expression of COL10A1 (I), GREM1(II), MMP11(III), POSTN(IV) and SPP1(V) regarding their associations with overall survival in LUAD patients. (B) Kaplan–Meier survival analysis conducted with high and low expression of COL10A1 (I), GREM1 (II), MMP11 (III), POSTN (IV) and SPP1 (V) regarding their associations with overall survival in LUSC patients. The number of patients in the high and low expression groups (n(high) and n(low)), Log-rank *P*-values, and HR with their associated *P*-values (p(HR)) are provided for each plot. The curves display the percentage of survival over time in months, with dashed lines representing the 95% confidence intervals. HR = hazard ratio, LUAD = lung adenocarcinoma, LUSC = lung squamus cell carcinoma.

To assess how well the 5 hub genes (COL10A1, POSTN, SPP1, MMP11, and GREM1), identified from the combined PPI network, predict patient outcomes, we conducted Kaplan–Meier survival analyses in LUAD and LUSC cohorts using the GEPIA2 web tool. These genes were selected based on their centrality in the shared COPD-LUAD-LUSC PPI network and known involvement in extracellular matrix (ECM) remodeling and tumor progression. According to log-rank tests performed using GEPIA data, high mRNA levels of SPP1 were significantly linked to improved overall survival in patients with LUAD, showing a hazard ratio of 1.4 and a *P*-value of 0.015. Similarly, in LUSC patients, elevated mRNA expression of GREM1 (hazard ratio 1.4, *P* = .03) and POSTN (hazard ratio 1.4, *P* = .013) were significantly correlated with better overall survival (Fig. 9A and B).

## 4. Discussion

Few studies have examined NSCLC and COPD combined, and it remains unknown how these 2 conditions are related. This study offers a thorough bioinformatic analysis of the shared molecular mechanisms shared by COPD and the 2 major subtypes of NSCLC: LUSC and LUAD. By integrating gene expression datasets and constructing regulatory networks, we identified key DEGs, TFs, and miRNAs that drive disease progression and represent promising targets for therapy. Our study offers new information on possible biomarkers and treatment targets that could be leveraged for early diagnosis and drug repurposing. Unlike prior studies such as Liao et al.,^[12]^ our integrative approach combines gene expression data, TF-miRNA-DEG networks, functional enrichment, and drug repurposing, offering broader mechanistic insights.

The identification of 15 common DEGs among COPD, LUSC, and LUAD underscores the significant molecular interplay between these diseases. COL10A1, POSTN, SPP1, MMP11, and GREM1 were the top 5 hub genes in the PPI network. These genes are linked to the remodeling of the extracellular matrix (ECM), immune regulation, and tumor progression. Functional classification of these DEGs further confirmed their roles in pathways such as cell adhesion, inflammation, and transcriptional regulation, highlighting their relevance in the pathophysiology of lung cancer and COPD. In LUAD, COL10A1 is elevated and associated with poor prognosis. Its overexpression promotes tumor progression by remodeling the ECM and activating the focal adhesion kinase signaling pathway via DDR2, enhancing cancer cell migration and survival.^[23]^ Although direct links to COPD are limited, similar ECM remodeling in both diseases suggests COL10A1 may contribute to a pro-tumorigenic microenvironment in COPD.

Periostin contributes to airway remodeling and fibrosis in COPD^[24]^ and is overexpressed in several cancers, including NSCLC. Its elevated levels are linked to poor prognosis, larger tumors, and advanced stages due to its role in angiogenesis, invasion, and metastasis. Periostin promotes tumor progression by interacting with integrin receptors and activating survival pathways.^[25,26]^ In COPD, chronic inflammation upregulates periostin, leading to ECM remodeling that may foster a tumor-promoting microenvironment, bridging COPD and lung cancer risk.

SPP1 is overexpressed in lung cancer and promotes cell invasion, migration, and tumor growth by upregulating COL11A1.^[27]^ In COPD, elevated SPP1 levels correlate with disease severity and drive chronic inflammation by recruiting immune cells.^[28]^ This persistent inflammation and ECM remodeling may create a tumor-promoting environment, linking COPD to increased lung cancer risk.

MMP11 is highly expressed in LUAD and promotes tumor development, invasion, and ECM remodeling through autocrine signaling. It is a potential therapeutic target, as antibody inhibition can reduce tumor growth.^[29]^ In COPD, MMP11-driven ECM changes may foster a pro-tumorigenic environment, linking it to increased lung cancer risk.

Elevated GREM1 levels are observed in advanced stages of COPD and are associated with how severe the disease is. It also plays a significant role in LUAD, where its inhibition may suppress tumor growth and metastasis.^[30,31]^ GREM1 deregulation in COPD contributes to a pro-fibrotic, pro-inflammatory environment that may promote lung cancer development.

Subgroup analysis revealed distinct gene expression patterns between COPD-LUSC and COPD-LUAD, with 13 and 29 common DEGs, respectively. Several genes (e.g., FLRT3, ID4, NR4A2) were downregulated in LUSC but upregulated in COPD and LUAD, while others (e.g., MMP11, SPP1, COL10A1) showed the opposite trend. These contrasting patterns suggest subtype-specific regulatory mechanisms. COPD-LUAD showed a more interconnected regulatory network, supporting greater molecular similarity with COPD and aligning with clinical data linking LUAD more frequently to COPD.

In COPD, LUSC, and LUAD, BP enrichment revealed pathways related to ECM organization, cell morphogenesis, and collagen fibril formation, highlighting roles for inflammation and ECM remodeling. LUAD showed additional enrichment in cancer-related pathways like cell adhesion, p53 signaling, and apoptosis. In contrast, LUSC was primarily linked to phospholipid transport, indicating less molecular overlap with COPD compared to LUAD.

GO terms within the CC category, such as “extracellular matrix” and “collagen-containing ECM,” highlight their importance in COPD and both lung cancer subtypes. In contrast, terms like “endoplasmic reticulum” and “plasma membrane” reflect intracellular differences, particularly between COPD and LUSC.

In COPD-LUAD, KEGG pathways related to cancer progression – such as “focal adhesion,” “leukocyte migration,” “viral carcinogenesis,” and “general cancer pathways” – were exclusively enriched. In contrast, COPD-LUSC showed no distinct KEGG enrichment, suggesting a focus on lipid and immune metabolism rather than cancer-specific mechanisms. The enrichment of viral carcinogenesis in COPD-LUAD points to a stronger link between viral infections and LUAD development compared to LUSC.

LUAD and COPD share enriched pathways in apoptosis, cell adhesion, and cancer progression, indicating stronger molecular ties. In contrast, LUSC and COPD overlap mainly in phospholipid transport and membrane structure. ECM remodeling and inflammation are prominent in LUAD but relevant to all 3. These findings highlight distinct molecular patterns: LUAD is linked to tumor-related signaling, while LUSC emphasizes immune and lipid metabolism. Understanding these differences may guide more targeted therapies for COPD-related lung cancer.

SP1 emerged as a key transcriptional regulator of shared DEGs in COPD, LUSC, and LUAD, interacting with genes like SPP1, SLC39A8, ID4, MMP11, and EDNRB. As noted by Beishline et al,^[32]^ SP1 regulates genes that play roles in differentiation, programmed cell death, and cell growth and division, and is critically involved in the advancement of lung cancer.^[33]^ It is also linked to COPD pathophysiology and has been identified as a shared regulatory node in lung cancer and emphysema. According to Karakioulaki et al,^[34]^ SP1 could potentially affect inflammation and the restructuring of tissues in COPD. Its dual role suggests SP1 might serve as a molecular link connecting COPD and lung cancer, where chronic inflammation in COPD may trigger abnormal SP1 activation, promoting tumor development.

The regulatory networks of LUSC and COPD showed less overlap, suggesting more distinct transcriptional regulation in LUSC. In contrast, the strong LUAD-COPD network indicates shared transcriptional processes and greater molecular similarity. This aligns with epidemiological data showing a closer association between LUAD and COPD than between LUSC and COPD.

miRNA-DEG interaction analysis identified “hsa-miR-335-5p,” “hsa-miR-192-5p,” and “hsa-miR-215-5p” as key regulators of multiple DEGs in COPD, LUSC, and LUAD, underscoring their role in disease progression. In NSCLC, “hsa-miR-335-5p” is downregulated and functions as a tumor suppressor by acting on oncogenes involved in cell cycle control and apoptosis, thereby inhibiting invasion and proliferation.^[35]^ Although direct studies on “hsa-miR-335-5p” in COPD are limited, it regulates pathways relevant to inflammation and tissue remodeling – central to COPD pathology. The ongoing inflammation characteristic of COPD may promote a tumor-prone microenvironment. “hsa-miR-192-5p” has also been demonstrated to act as a tumor suppressor in NSCLC,^[36]^ while “hsa-miR-215-5p” shows a significant decrease in expression in NSCLC and suppresses tumor progression in several cancers.^[37]^ Dysregulation of these miRNAs may alter gene expression related to inflammation, cell proliferation, and apoptosis, linking COPD with lung cancer development.

In COPD-LUSC networks, “hsa-miR-335-5p” and **“**hsa-miR-6867-5p” were identified, indicating subtype-specific miRNA regulation. In contrast, “hsa-miR-16-5p,” “hsa-miR-17-5p,” **“**hsa-miR-26b-5p,” and “hsa-miR-221-3p” were enriched in both COPD and LUAD, supporting a stronger molecular link between these 2 conditions.

miRNA-DEG-TF network analysis revealed NR4A2 as the most frequently targeted gene, regulated by multiple miRNAs and TFs, suggesting its role as a key molecular hub linking COPD and lung cancer. Major TFs SP1 and CTCF were also identified, highlighting complex transcriptional and post-transcriptional regulation in COPD–lung cancer progression.

Survival analysis showed that high expression of SPP1 (*P* = .015) was linked to better outcomes in LUAD, while POSTN (*P* = .013) and GREM1 (*P* = .03) were associated with improved prognosis in LUSC. These findings suggest that SPP1, POSTN, and GREM1 may serve as context-dependent biomarkers for both risk assessment and therapeutic targeting in LUAD and LUSC.

Medication-gene interaction analysis identified several promising drug repurposing candidates for COPD, LUSC, and LUAD. Minocycline (anti-inflammatory), marimastat (MMP inhibitor), and bosentan (endothelin receptor antagonist) target shared molecular pathways. Sitaxentan, used for PAH, may also lower COPD-related lung cancer risk. Marimastat may further inhibit tumor angiogenesis and metastasis. Subtype-specific candidates include octreotide and pasireotide targeting SSTR1 (COPD-LUSC), and tacrine and choline targeting BCHE (COPD-LUAD). These findings support potential pharmacological strategies to reduce lung cancer risk in COPD patients.

This study has some limitations, even though it provides insightful information. In vitro and in vivo research is required for experimental validation because the use of publicly accessible datasets increases uncertainty in sample collection and processing. To improve their clinical applicability, future studies should focus on confirming these indicators and treatment targets in separate patient groups. These results open the door for further translational research and customized medicine strategies by highlighting possible biomarkers for early detection and treatment options for focused therapy. Finally, this study establishes a foundation for tailored treatment strategies to enhance the prognosis of COPD patients who are susceptible to lung cancer.

## 5. Conclusion

This study not only deepens our understanding of the pathogenic interplay between COPD and lung cancer, but also proposes novel molecular targets and therapeutic avenues for mitigating the increased cancer risk in patients with COPD. These insights lay the groundwork for personalized medical approaches, improved diagnostic strategies, and targeted treatments for the management of COPD-associated NSCLC.

## Author contributions

**Conceptualization:** Sevinç Akçay.

**Data curation:** Sevinç Akçay.

**Formal analysis:** Sevinç Akçay.

**Investigation:** Sevinç Akçay.

**Methodology:** Sevinç Akçay.

**Project administration:** Sevinç Akçay.

**Resources:** Sevinç Akçay.

**Software:** Sevinç Akçay.

**Supervision:** Sevinç Akçay.

**Validation:** Sevinç Akçay.

**Visualization:** Sevinç Akçay.

**Writing – original draft:** Sevinç Akçay.

**Writing – review & editing:** Sevinç Akçay.

## Supplementary Material


