# Supplementary material for: Deciphering molecular overlaps between COPD and NSCLC subtypes (LUAD and LUSC): An integrative bioinformatics study
Source: Medicine (Baltimore). 2025 Aug 15;104(33):e43906. doi: 10.1097/MD.0000000000043906 (PMC12366952; doi:10.1097/MD.0000000000043906)
Supplement: Supplementary file 1 [file medi-104-e43906-s001.pdf]

Supplementary Table 1. Full List of Common DEGs between COPD and LUSC

Gene symbol	COPD		LUSC	
	***P*-value**	**Log2FC**	***P*-value**	**Log2FC**
AGR3	2.80e-06	2.14004	6.32e-12	-1.42927
AKAP12	9.53e-05	1.77750	8.57e-16	-1.06023
ATP11B	3.50e-04	1.34325	1.13e-22	1.03134
FNDC1	1.55e-03	1.12869	1.89e-19	1.41170
GIMAP1	2.74e-03	1.11462	8.09e-27	-1.0207
HPGDS	3.39e-05	1.06122	2.88e-26	-1.16889
NTN4	6.11e-05	1.57835	7.12e-18	-1.06565
SLC4A4	1.76e-06	1.51954	1.31e-14	-1.03422
SOX7	4.31e-05	1.11404	2.47e-15	-1.11436
SSTR1	2.53e-03	1.13093	4.94e-23	-1.26653
VAPA	1.98e-03	1.27615	1.30e-32	-1.13267
ADAMDEC1	5.28e-03	-1.06344	3.90e-15	1.40123
DAPL1	9.19e-05	-1.36083	1.93e-09	1.17507

COPD = chronic obstructive pulmonary disease, LUSC = lung squamous cell carcinoma.

Supplementary Table 2. Full List of Common DEGs between COPD and LUAD

Gene symbol	COPD		LUAD	
	***P*-value**	**Log2FC**	***P*-value**	**Log2FC**
LSM5	3.17e-06	1.82151	8.64e-18	-1.02384
BCHE	1.67e-07	1.17073	2.41e-26	1.65197
CAT	1.78e-05	1.30778	3.61e-32	1.37352
DNAJB4	6.81e-06	1.69741	1.46e-16	1.02348
EPB41L2	3.64e-05	1.62403	9.24e-24	1.09227
EPCAM	6.84e-05	1.47599	1.50e-19	-1.13451
FABP5	3.76e-05	1.79802	7.25e-17	1.52334
ITM2A	2.20e-05	1.84949	5.01e-28	1.83541
KAT2B	4.02e-05	1.34918	3.65e-17	1.00807
KCTD12	1.38e-03	1.34071	6.81e-14	1.00986
KPNA2	2.20e-06	1.61385	3.93e-22	-1.35426
LAMA4	2.47e-05	1.14597	7.01e-18	1.17709
METTL7A	1.40e-04	1.10346	1.28e-29	1.68517
NEDD9	2.92e-05	1.13609	4.01e-17	1.46499
P2RY14	1.90e-08	1.68428	1.35e-24	1.49217
PERP	2.63e-05	1.47146	7.30e-13	-1.13238
PIK3R1	2.87e-05	1.76949	1.55e-22	1.11803
PLA2G4A	1.29e-05	1.35541	6.56e-08	-1.23998
PLAGL1	2.59e-06	1.69407	5.59e-15	1.28968
PMAIP1	1.43e-04	1.38993	8.77e-13	-1.49243
SORD	5.33e-05	1.42188	1.58e-18	-1.32023
TFPI2	3.44e-06	1.39947	9.21e-06	-1.36757
TMEM106B	2.89e-03	1.32074	1.23e-18	-1.23921
ZEB2	1.74e-04	1.16521	1.66e-16	1.14814
COMP	5.86e-05	-1.47814	3.00e-11	-1.33701
CXCL12	2.14e-07	-1.05979	1.32e-13	-2.05430
MMP9	6.94e-05	-1.25618	1.15e-14	-1.40508
POU2AF1	9.80e-07	-1.32888	3.35e-08	-1.24239
SRPX2	7.95e-09	-1.02034	3.32e-12	-1.02360

COPD = chronic obstructive pulmonary disease, LUAD = lung adenocarcinoma.

Supplementary Table 3. Transcription Factors (TFs) Regulating Shared DEGs in COPD, LUAD, and LUSC

Transcription factor	Number of target genes	Associated group(s)
SP1	5	COPD, LUAD, LUSC
SP1	4	COPD, LUAD
NFKB1	4	COPD, LUAD
KLF6	3	COPD, LUAD
TP53	3	COPD, LUAD
RELA	3	COPD, LUAD
PPARG	2	COPD, LUAD
SNAI2	2	COPD, LUAD
ESR1	2	COPD, LUAD
HDAC1	2	COPD, LUAD

COPD = chronic obstructive pulmonary disease, LUSC = lung squamous cell carcinoma, LUAD = lung adenocarcinoma.

Supplementary Table 4. Transcription factors (TFs) regulating shared DEGs in COPD, LUAD, and LUSC in TF-miRNA-gene network

Transcription factor	Number of target genes	Associated group(s)	Transcription factor	Number of target genes	Associated group(s)
SP1	3	COPD, LUAD, LUSC	STAT1	4	COPD, LUAD
CTCF	3	COPD, LUAD, LUSC	POU2F1	4	COPD, LUAD
RREB1	2	COPD, LUAD, LUSC	EGR1	4	COPD, LUAD
TFAP2C	2	COPD, LUAD, LUSC	SPI1	4	COPD, LUAD
TFAP2A	2	COPD, LUAD, LUSC	HNF1A	3	COPD, LUAD
CEBPA	2	COPD, LUAD, LUSC	EBF1	3	COPD, LUAD
USF1	2	COPD, LUAD, LUSC	RELA	3	COPD, LUAD
CUX1	2	COPD, LUAD, LUSC	PAX5	3	COPD, LUAD
CREB1	2	COPD, LUAD, LUSC	AR	3	COPD, LUAD
CDC5L	2	COPD, LUAD, LUSC	NFYA	3	COPD, LUAD
ATF6	2	COPD, LUAD, LUSC	GABPA	3	COPD, LUAD
MYC	2	COPD, LUAD, LUSC	TCF3	3	COPD, LUAD
ATF2	2	COPD, LUAD, LUSC	STAT2	3	COPD, LUAD
MIF	2	COPD, LUAD, LUSC	BACH1	3	COPD, LUAD
HNF1A	2	COPD, LUAD, LUSC	POU2F2	3	COPD, LUAD
SMAD4	2	COPD, LUAD, LUSC	SMAD1	3	COPD, LUAD
TBP	2	COPD, LUAD, LUSC	SMAD2	3	COPD, LUAD
STAT3	2	COPD, LUAD, LUSC	TFAP4	3	COPD, LUAD
FOXC1	2	COPD, LUAD, LUSC	ZBTB6	3	COPD, LUAD
MEIS1	2	COPD, LUAD, LUSC	E2F1	3	COPD, LUAD
SMAD1	2	COPD, LUAD, LUSC	MAX	3	COPD, LUAD
NKX3-1	2	COPD, LUAD, LUSC	ETV4	3	COPD, LUAD
POU2F1	2	COPD, LUAD, LUSC	NFKB1	3	COPD, LUAD
EGR1	4	COPD, LUSC	HNF4A	3	COPD, LUAD
USF1	3	COPD, LUSC	MXI1	3	COPD, LUAD
EGR3	3	COPD, LUSC	IRF1	2	COPD, LUAD
E2F1	2	COPD, LUSC	CREB1	2	COPD, LUAD
POU2F1	2	COPD, LUSC	P2RY	2	COPD, LUAD
CTCF	2	COPD, LUSC	NFE2L1	2	COPD, LUAD
SREBF2	2	COPD, LUSC	GATA3	2	COPD, LUAD
SREBF1	2	COPD, LUSC	TAL1	2	COPD, LUAD
CREB1	2	COPD, LUSC	RELB	2	COPD, LUAD
HPGDS	2	COPD, LUSC	MEF2A	2	COPD, LUAD
EGR2	2	COPD, LUSC	GATA1	2	COPD, LUAD
SP1	2	COPD, LUSC	MEIS1	2	COPD, LUAD
JUND	2	COPD, LUSC	HOXA9	2	COPD, LUAD
JUNB	2	COPD, LUSC	CTBP1	2	COPD, LUAD
JUN	2	COPD, LUSC	NFE2	2	COPD, LUAD
FOSL1	2	COPD, LUSC	RORA	2	COPD, LUAD
FOSB	2	COPD, LUSC	CREBBP	2	COPD, LUAD
FOS	2	COPD, LUSC	EP300	2	COPD, LUAD
MXI1	2	COPD, LUSC	ELK1	2	COPD, LUAD
MAX	2	COPD, LUSC	ABL1	2	COPD, LUAD
TP53	7	COPD, LUAD	NFYC	2	COPD, LUAD
SP1	7	COPD, LUAD	NFYB	2	COPD, LUAD
JUN	7	COPD, LUAD	RARA	2	COPD, LUAD
CTCF	6	COPD, LUAD	ARNT	2	COPD, LUAD
SMAD3	6	COPD, LUAD	FOXJ2	2	COPD, LUAD
YY1	5	COPD, LUAD	SRF	2	COPD, LUAD
USF1	5	COPD, LUAD	FOS	2	COPD, LUAD
PPARG	5	COPD, LUAD	JUND	2	COPD, LUAD
TFAP2A	5	COPD, LUAD	JUNB	2	COPD, LUAD
MYC	5	COPD, LUAD	FOSL1	2	COPD, LUAD
ESR1	4	COPD, LUAD	FOSB	2	COPD, LUAD
STAT1	4	COPD, LUAD	FOXA2	2	COPD, LUAD

COPD = chronic obstructive pulmonary disease, LUSC = lung squamous cell carcinoma, LUAD = lung adenocarcinoma.

Supplementary Table 5. Key miRNAs identified in the TF-miRNA-gene regulatory network analysis

miRNA Name	Number of Target Genes	Associated Group(s)	miRNA Name	Number of Target Genes	Associated Group(s)
"hsa-miR-340"	5	COPD, LUAD, LUSC	"hsa-miR-144"	5	COPD, LUAD
"hsa-miR-144"	4	COPD, LUAD, LUSC	"hsa-miR-495"	5	COPD, LUAD
"hsa-miR-376c"	3	COPD, LUAD, LUSC	"hsa-miR-137"	5	COPD, LUAD
"hsa-miR-145"	3	COPD, LUAD, LUSC	"hsa-miR-200c"	5	COPD, LUAD
"hsa-miR-300"	3	COPD, LUAD, LUSC	"hsa-miR-181a"	5	COPD, LUAD
"hsa-miR-802"	3	COPD, LUAD, LUSC	"hsa-miR-19a"	4	COPD, LUAD
"hsa-miR-194"	3	COPD, LUAD, LUSC	"hsa-miR-19b"	4	COPD, LUAD
"hsa-miR-768-5p"	3	COPD, LUAD, LUSC	"hsa-miR-448"	4	COPD, LUAD
"hsa-miR-522"	3	COPD, LUAD, LUSC	"hsa-miR-26a"	4	COPD, LUAD
"hsa-miR-342"	3	COPD, LUAD, LUSC	"hsa-miR-26b"	4	COPD, LUAD
"hsa-miR-19a"	3	COPD, LUAD, LUSC	"hsa-miR-142-3p"	4	COPD, LUAD
"hsa-miR-101"	3	COPD, LUAD, LUSC	"hsa-miR-498"	4	COPD, LUAD
"hsa-miR-125a-3p"	2	COPD, LUAD, LUSC	"hsa-miR-101"	4	COPD, LUAD
"hsa-miR-3183"	2	COPD, LUAD, LUSC	"hsa-miR-347b"	4	COPD, LUAD
"hsa-miR-944"	2	COPD, LUAD, LUSC	"hsa-miR-181b"	4	COPD, LUAD
"hsa-let-7a"	2	COPD, LUAD, LUSC	"hsa-miR-181c"	4	COPD, LUAD
"hsa-miR-30e"	2	COPD, LUAD, LUSC	"hsa-miR-181d"	4	COPD, LUAD
"hsa-miR-153"	2	COPD, LUAD, LUSC	"hsa-miR-135b"	4	COPD, LUAD
"hsa-miR-1"	2	COPD, LUAD, LUSC	"hsa-miR-93"	3	COPD, LUAD
"hsa-miR-217"	2	COPD, LUAD, LUSC	"hsa-miR-367"	3	COPD, LUAD
"hsa-miR-202"	2	COPD, LUAD, LUSC	"hsa-miR-524-5p"	3	COPD, LUAD
"hsa-miR-548a-5p"	2	COPD, LUAD, LUSC	"hsa-miR-96"	3	COPD, LUAD
"hsa-miR-145"	4	COPD, LUSC	"hsa-miR-612"	3	COPD, LUAD
"hsa-miR-410"	3	COPD, LUSC	"hsa-miR-139-5p"	3	COPD, LUAD
"hsa-miR-203"	3	COPD, LUSC	"hsa-miR-141"	3	COPD, LUAD
"hsa-miR-488"	2	COPD, LUSC	"hsa-miR-132"	3	COPD, LUAD
"hsa-miR-24"	2	COPD, LUSC	"hsa-miR-212"	3	COPD, LUAD
"hsa-miR-548a-3p"	2	COPD, LUSC	"hsa-miR-103"	3	COPD, LUAD
"hsa-miR-656"	2	COPD, LUSC	"hsa-miR-454"	2	COPD, LUAD
"hsa-miR-300"	2	COPD, LUSC	"hsa-miR-18a"	2	COPD, LUAD
"hsa-miR-381"	2	COPD, LUSC	"hsa-miR-520a-5p"	2	COPD, LUAD
"hsa-miR-127-5p"	2	COPD, LUSC	"hsa-miR-155"	2	COPD, LUAD
"hsa-miR-217"	2	COPD, LUSC	"hsa-miR-153"	2	COPD, LUAD
"hsa-miR-520d-5p"	2	COPD, LUSC	"hsa-miR-186"	2	COPD, LUAD
"hsa-miR-524-5p"	2	COPD, LUSC	"hsa-miR-218"	2	COPD, LUAD
"hsa-miR-222"	6	COPD, LUAD	"hsa-miR-25"	2	COPD, LUAD
"hsa-miR-548c-3p"	6	COPD, LUAD	"hsa-miR-32"	2	COPD, LUAD
"hsa-miR-221"	5	COPD, LUAD	"hsa-miR-603"	2	COPD, LUAD

COPD = chronic obstructive pulmonary disease, LUSC = lung squamous cell carcinoma, LUAD = lung adenocarcinoma.
